# Establishment and Phenotypic Analysis of the Novel Gaucher Disease Mouse Model With the Partially Humanized *Gba1* Gene and F213I Mutation

**DOI:** 10.3389/fgene.2022.892457

**Published:** 2022-05-27

**Authors:** Jia-ni Guo, Ming Guan, Nan Jiang, Na Li, Ya-jun Li, Jin Zhang, Duan Ma

**Affiliations:** ^1^ Key Laboratory of Metabolism and Molecular Medicine, Ministry of Education, Department of Biochemistry and Molecular Biology, School of Basic Medical Sciences, Shanghai Medical College, Fudan University, Shanghai, China; ^2^ Huashan Hospital, Fudan University, Shanghai, China; ^3^ Children’s Hospital, Fudan University, Shanghai, China

**Keywords:** Gaucher disease, *GBA1* gene, F213I mutation, GD mouse model, partially humanized

## Abstract

Gaucher disease (GD) is an autosomal recessive lysosomal storage disorder caused by mutations in the *GBA1* gene, which produces the glucocerebrosidase (GCase) protein. There are more than 500 mutations reported in *GBA1*, among which L444P (p.Leu444Pro) and F213I (p.Phe213Ile) are the most common in the Chinese population, while the function of F213I mutation remains elusive. This study aims to establish the GD mouse model of partially humanized *Gba1* gene with F213I mutation. *In vitro* GCase activity assays showed that the product of partially humanized *Gba1* gene, in which the mouse exons 5-7 were replace by the corresponding human exons, displayed similar activity with the wild-type mouse *Gba1*, while the F213I mutation in the humanized *Gba1* led to significant decrease in enzyme activity. ES cell targeting was used to establish the mice expressing the partially humanized *Gba1*-F213I. *Gba1* ^F213I/+^ mice did not show obviously abnormal phenotypes, but homozygous *Gba1* ^F213I/F213I^ mice died within 24 h after birth, whose epidermal stratum corneum were abnormal from the wild-type. The GCase activity in *Gba1* ^F213I/F213I^ mice greatly decreased. In conclusion, our results showed that the partially humanized GD mouse model with the F213I mutation was developed and homozygous F213I mutation is lethal for newborn mice.

## Introduction

Gaucher disease (GD) is one of the most common lysosomal storage diseases. GD is an autosomal recessive hereditary disease caused by mutations in the gene encoding β- Glucocerebrosidase (*GBA1*). Due to the deficiency of glucocerebrosidase (GCase) activity, glucosylceramide (GlcCer) accumulates in the lysosomes and is metabolized to produce glucosylsphingosine, sphingosine and then sphingosine-1-phosphate (S1P) (Stirnemann et al., 2017). GD is characterized by enlargement of liver and spleen, lesions in the bones, and, in the most severe cases, neuropathology accompanied by neuroinflammation (Dasgupta et al., 2015; Do et al., 2019; Oguri et al., 2020). According to the impairment of central nervous system function, there are three GD types (Carubbi et al., 2020). Gaucher disease type 1 (GD1) was regarded as the mildest form without obvious neurologic involvement at an early stage, but some GD1 patients developed Parkinson disease phenotype at older age (Kartha et al., 2020). Type 2 is the most severe form and appears as an early onset of neurologic disease with an acute course. Type 3 disease is of intermediate severity with a later onset of neurologic symptoms and a more chronic course (Kartha et al., 2020).

*GBA1* is located in human chromosome 1q21-22, 7.2 kb long, composed of 11 exons and 10 introns. More than 500 types of mutations linked to GD have been found in *GBA1*, including splice site mutation, point mutation, coding frameshift mutation, insertion or deletion mutation (Milenkovic et al., 2022). The clinical features of GD are dictated to a large extent by mutation patterns carried in the *GBA1* gene. In China, the most common mutations in *GBA1* include L444P (p.Leu444Pro, 33.00%), F213I (p.Phe213Ile, 5.33%) and N188S (p.Asn188Ser, 5.33%) (Zhang et al., 2009). F213I mutation, the A-to-T transversion at nt 754 in exon 6 (NM_000157.4: c.754T > A), is also named F252I (p.Phe252Ile) according to the new nomenclature and is the second common point mutant *GBA1* allele in Chinese GD patients (He et al., 1992; Zhang et al., 2009; Oto et al., 2021). The F213I mutation was found in all three types of GD, and F213I-associated types 2 GD and type 3 GD were more prevalent in Asian populations (Koprivica et al., 2000; Tajima et al., 2009; Zhang et al., 2009; Vieira and Schapira, 2021).

Mouse models are widely used in GD research. Several mouse models with common *Gba1* mutations in GD patients have been established, such as L444P mice, D409V and N370S mice (Liu et al., 1998; Xu et al., 2003; Jackson et al., 2019; Liou et al., 2019; Migdalska-Richards et al., 2020). However, up to date, there have been no investigation on the F213I mutation in mice. Furthermore, researches on conserved genes have shown that human-mouse chimeric gene can function normally and, based on this finding, exons of mouse genes could be replaced by human counterparts to generate partially humanized mouse model, which will be suitable to detect effectiveness of human genome-editing therapeutic methods *in vivo* in mice (Dong et al., 2012; Takeuchi et al., 2019; Guo et al., 2021). In this study, a GD mouse model with partially humanized F213I *Gba1* was established.

## Materials and Methods

### Mice and Genotyping

The Institutional Animal Care and Use Committee of Fudan University, China approved all protocols. Mice with partially humanized *Gba1* F213I allele (mh*Gba1*-F213I) were generated by using the ES-cell-based gene targeting technology at Shanghai Model Organisms Center in Shanghai, China. Briefly, the ES cell targeting vector was constructed by fusions, containing 3.0 kb 5′ homologous arm, h*GBA1* exon 5-7 with F213I mutation, PGK-Neo-poly A, 3.0 kb 3′ homologous arm and MC1-TK-polyA, a negative screening marker. The vector was linearized and transferred into JM8A3 ES cell by electroporation. After PCR identification, the positive ES cell clones were amplified and injected into the blastocysts of C57BL/6J mice to obtain chimeric mice. The Neo-removed *Gba1* ^+/F213I^ mice were obtained by mating chimeric mice with mice with *Flp* gene. In this way, the exon 5-7 site of the mouse *Gba1* gene was replaced by the human *GBA1* exon 5–7. The genotype of each mouse was determined by PCR analysis of genomic DNA prepared from tail biopsies. PCR was performed by using the forward primer m*Gba1*-F and the reverse primer m*Gba1*-T for wild type or the forward primer h*GBA1*-F and the reverse primer h*GBA1*-T for mutants. The 5′ homology arm and the 3′ homology arm were amplified for sequencing. Sequencing was performed by the Tsingke Biotechnology Co., Ltd. in China. Primer m*Gba1*-5F and h*GBA1*-5T were designed for 5′ homology arm, while h*GBA1*-3F and m*Gba1*-3T were designed for 3’ homology arm. The primers are listed in Supplementary Table S1.

### mRNA Extraction and qRT-PCR

Total RNA was extracted from tissues using TRIzol reagent (Thermo Fisher) according to the manufacturer’s protocol. RNA degradation and contamination were assessed on 1% agarose gels, and the RNA concentration was measured by using a NanoDrop 1,000 spectrophotometer (Thermo Scientific). cDNA was synthesized by using the Hifair III 1st Strand cDNA Synthesis SuperMix for qPCR (gDNA digester plus; Yeasen), and the integrity of the synthesized cDNA was confirmed by using glyceraldehyde 3-phosphate dehydrogenase (Gapdh) as the endogenous control. Real-time PCR was carried out using SYBR Premix Ex Taq TM II (Perfect Real Time; TaKaRa) and measured by using an ABI 7500 instrument. qRT-PCR was performed by using the forward primer m*Gapdh*-RF and the reverse primer m*Gapdh*-RT for *Gapdh* or the forward primer m*Gba1*-RF and the reverse primer m*Gba1*-RT for *Gba1*. The primers are listed in Supplementary Table S1. PCR was performed as reported by Zhang et al. (Zhang et al., 2019).

### GCase Expression *in vitro*

Crispr/Cas9 system was used to reduce the interference of the endogenous GCase activity, as we performed previously (Abbasi et al., 2020). Briefly, HEK293 cells were infected with lentivirus expressing CAS9 protein and sgRNA of *GBA1.* Infected cells were pooled by using puromycin selection (1ug/ml), and after 7 days, the cells were conducted for other assays. Partially humanized cDNA was constructed by Tsingke Biotechnology Co., Ltd. Single mutagenesis was inserted by overlapping PCR. Wild type mouse cDNA was synthesized by taking mRNA from wild type mice as template. The sequences are listed in Supplementary Table S2. All types of cDNA were subcloned into the PCDH- immediate early enhancer and promoter (CMV)-HA plasmid. Plasmids were transferred into HEK293 cells whose *GBA1* was knocked down by CRISPR-Cas9. Cells were harvested for following GCase activity assays and western blotting forty-eight hours after the transient transformation.

### GCase Enzyme Activity Assay

The GCase enzyme activity assay on the homogenate samples including sample preparation was performed according to the manufacturer’s instructions of Glucosylceramidase Activity Assay Kit (Fluorometric; BioVision). Fluorescence intensity (Ex/Em = 360/445 nm) was detected in a Multimode Plate Reader (PerkinElmer, EnSpire). During the measurement, blank controls without GCase were set to remove background value. According to the manufacturer’s instructions, specific sample Gucosylceramidase activity = B/(30 × V × P) × D = pmol/min. mg ≡ µU/mg, where B is 4-MU amount from the standard curve (pmol), 30 is the reaction time (min), V is sample volume added into the reaction well (ml), P is initial sample concentration in mg-protein/ml (mg/ml), D is sample dilution factor. One unit of Glucosylceramidase activity is the amount of enzyme that generates 1.0 µmol of 4-Methylumbelliferone per min at pH 4.5 at 37°C. The weight of each mouse tissue has been carefully weighed, and the relative quantity of GCase per mg tissue was calculated, respectively.

### Western Blot Analysis

Protein extracts from cells were prepared using a lysis buffer (200 mM Tris-HCl [pH 7.5], 1.5 M NaCl, 10 mM EDTA, 10 mM EGTA, 25 mM sodium pyrophosphate, 10 mM β-glycerophosphate, 1 mM Na3VO4, 50 mM NaF) supplied with a protease inhibitor cocktail (Roche Diagnostics). Protein samples of 20 ug each sample were separated on a 10% polyacrylamide gel and analyzed by Western blot using anti-HA (Proteintech, 51064-2-AP, 1:3,000) and anti-Gapdh (Proteintech, 10494-1-AP, 1:1,000) antibodies. Peroxidase-conjugated rabbit immunoglobulin G (IgG; Jackson ImmunoResearch, 1:2,500) was used as the secondary antibody. Western blots were developed using ImmobilonTM Western Chemiluminescent HRP Substrate (Merck Millipore), and analysis was performed with a Luminescent Image Analyzer (GE, ImageQuant LAS 4000 mini). The results were quantified by using the ImageJ software.

### Histological Analysis

Tissues were immersion fixed with 4% neutral-buffered paraformaldehyde, embedded tissues in paraffin blocks and prepared 5 µm sections. Sections of the different tissues were stained with hematoxylin and eosin as reported previously (Xiao et al., 2017; Du et al., 2019). Sections of skin were also stained with period acid-Schiff as reported (Sun et al., 2020).

### Statistical Analysis

Statistical significance was assessed using Student’s *t*-test, as reported in the figure legends. The results were significant at *p* values under 0.05. All statistical tests were performed using Prism software (GraphPad, version 8.0).

## Results

### F213I Point Mutation in Partially Humanized *GBA1* Gene Led to Decreased GCase Activity

**FIGURE 1:**
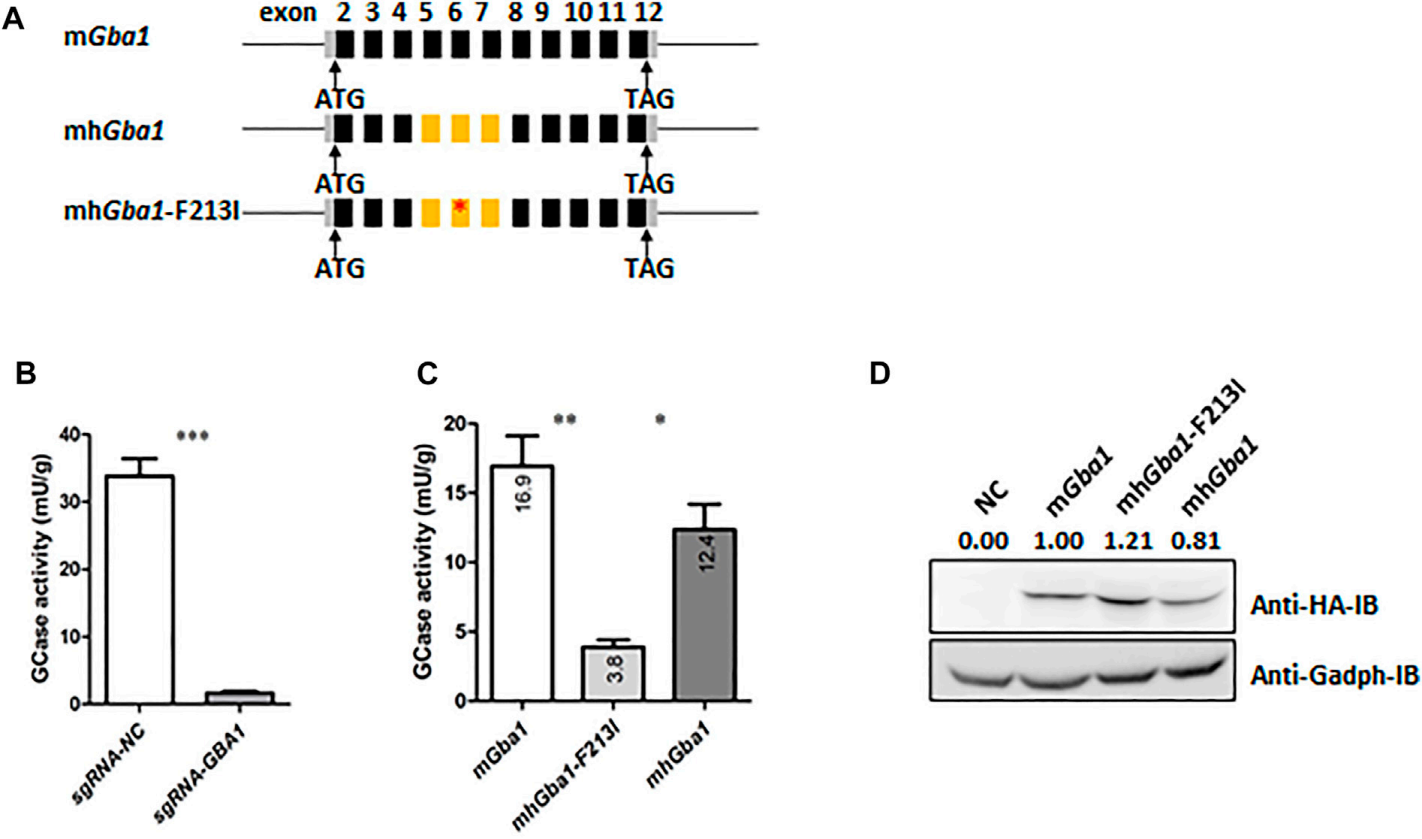
F213I point mutation in partially humanized *Gba1* gene led to decreased GCase activity *in vitro*. **(A)** Structures of the mh*Gba1*-WT and mh*Gba1*-F213I expression plasmids. Exons 5-7 of mouse *Gba1* cDNA are replaced by exons 5-7 of human *GBA1*. F213I mutation was introduced into the partially humanized *Gba1* cDNA. Black bars represent mouse *Gba1* coding exons. Grey bars represent *Gba1* uncoding exons. Lines between bars represent introns. Yellow bars represent human *GBA1* coding exons, and red asterisk represents F213I mutation site. **(B)** GCase activity was detected in the Crispr/Cas9-mediated *GBA1*-knockdown human HEK293 cells, using normal human HEK293 cells as control. To reduce interference of the endogenous GCase activity, the human HEK293 cells were infected with lentivirus expressing CAS9 protein and sgRNA of *GBA1*, pooled by using puromycin selection (1 ug/ml) for 7 days and were collected for the GCase activity assays. The results were expressed as the mean—SEM and difference between groups was analyzed by Student’s t-test, ***p < 0.001. **(C)** The endogenous *GBA1-*knocked down human HEK293 cells were transfected respectively with the mh*Gba1*-WT, mh*Gba1*-F213I and m*Gba1*-WT expression plasmids, and collected for GCase activity assays 48 h after transfection. The results were expressed as the mean—SEM and differences between every two group were analyzed by Student’s *t*-test, *p < 0.05, **p < 0.01. **(D)** Western blot was used to detect the expression of the different types of HA-tagged GCase, with Gapdh as an internal reference. Results were quantified with the ImageJ software, and the relative expression values were labelled, taking the mGba1 as 100%.

The *GBA1* gene is highly conserved in mice and humans. Both of them have 11 exons and exons matched one-to-one (had the same boundaries in both genomes), and matching encoding exons are highly similar (84% sequence identity). F213I, the second popular point mutant *GBA1* allele in Chinese GD patients, lies in the exon 6 of human and mouse *GBA1* genes. In order to establish the GD mouse model with partially humanized *Gba1* gene carrying F213I mutation, we planned to replace mouse *Gba1* exons 5-7 with human exons 5-7 carrying the F213I (Figure 1A). First, we detected the effects of the partial humanization on the activity of GCase. In order to exclude the interference of the endogenous GCase activity, the *GBA1* gene of human HEK293 cells was knocked down by using Crispr/Cas9 system (Figure 1B). The *GBA1*-knocked down HEK293 cells were transfected, respectively with the plasmids expressing mouse *Gba1* (m*Gba1*), partially humanized *Gba1* (mh*Gba1*) and mh*Gba1* with F213I (mh*Gba1*-F213I). GCase activity assays showed that there was no significant difference in GCase activity between m*Gba1* and mh*Gba1* while the activity of mh*Gba1*-F213I was greatly reduced (Figure 1C). Western botting was carried out to detect the expression of the different types of HA-tagged GCase (Figure 1D). Considering the relative values of GCase activity to protein amount, the activity of the mh*Gba*-F213I protein production was about 19% of the m*GBA,* while the activity of. mh*GBA* was similar to the m*Gba*.

### Establishment of GD Mouse Model With Partially Humanized *Gba1* Gene and F213I Point Mutation

**FIGURE 2:**
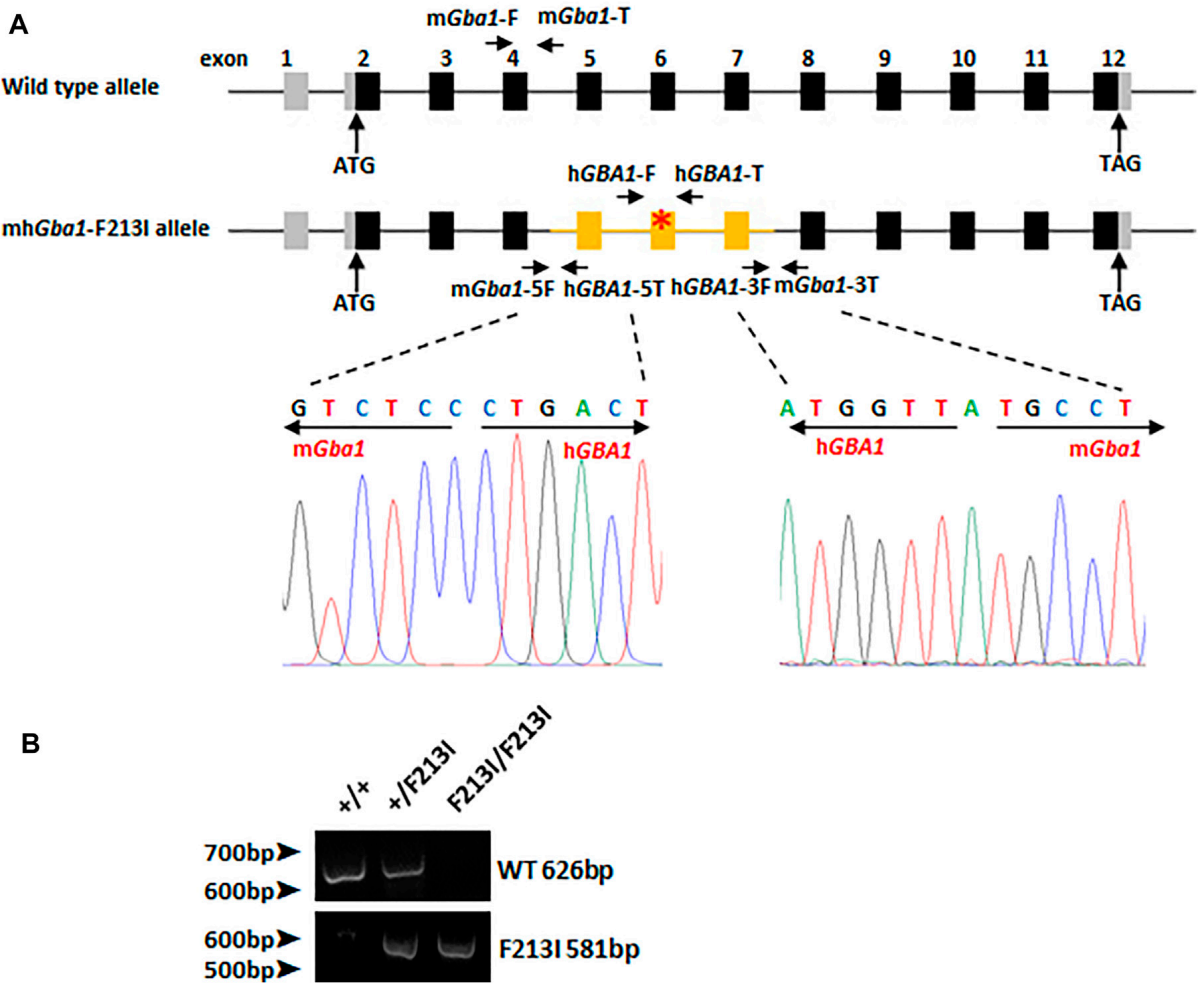
The construction of the mh*Gba1*-F213I mice. **(A)** A general scheme of the genome of wild type mice and mh*Gba1*-F213I mice. Black bars represent mouse *Gba1* coding exons. Grey bars represent *Gba1* uncoding exons. Yellow bars represent human *GBA1* coding exons, and red asterisk represents mutation site. The WT allele of *Gba1* was measured by using the m*Gba1*-F primer with the reverse m*Gba1*-T primer. The mh*Gba*-F213I allele was measured by using the h*GBA1*-F primer with the reverse h*GBA1*-T primer. Recombination sites were amplified by PCR and sequenced to confirm the homologous substitution. Primer m*Gba1*-5F and h*GBA1*-5T were used for upstream recombination boundary sequencing. Primer h*GBA1*-3F and m*Gba1*-3T were used for downstream recombination boundary sequencing. **(B)** PCR analysis of DNA extracted from tails of *Gba1* (+/+) mice, *Gba1* (F213I/+) mice and *Gba1* (F213I/F213I) mice for genotype identification.

By using gene targeting technology, the mouse *Gba1* genomic DNA fragment containing exons 5 to 7 were substituted by the human counterparts carrying the F213I mutation. The upstream and downstream recombination boundaries were validated by DNA sequencing, revealing that the mouse *Gba1* DNA fragment from exon 5 to 7 was correctly replaced with the humanized fragment (Figure 2A). Genomic PCR was used for genotyping (Figure 2B). *Gba1* ^+/F213I^ mice were obtained by crossing chimeric mice with wild-type mice and displayed no obvious abnormality. Up to now, we have not observed the symptoms of Parkinson disease in 6-month-old F213I heterozygotes. A lifespan observation of the F213I mice may be needed in future research.

### Early Postnatal Lethality in Mice With the Homozygous F213I Mutation

**FIGURE 3:**
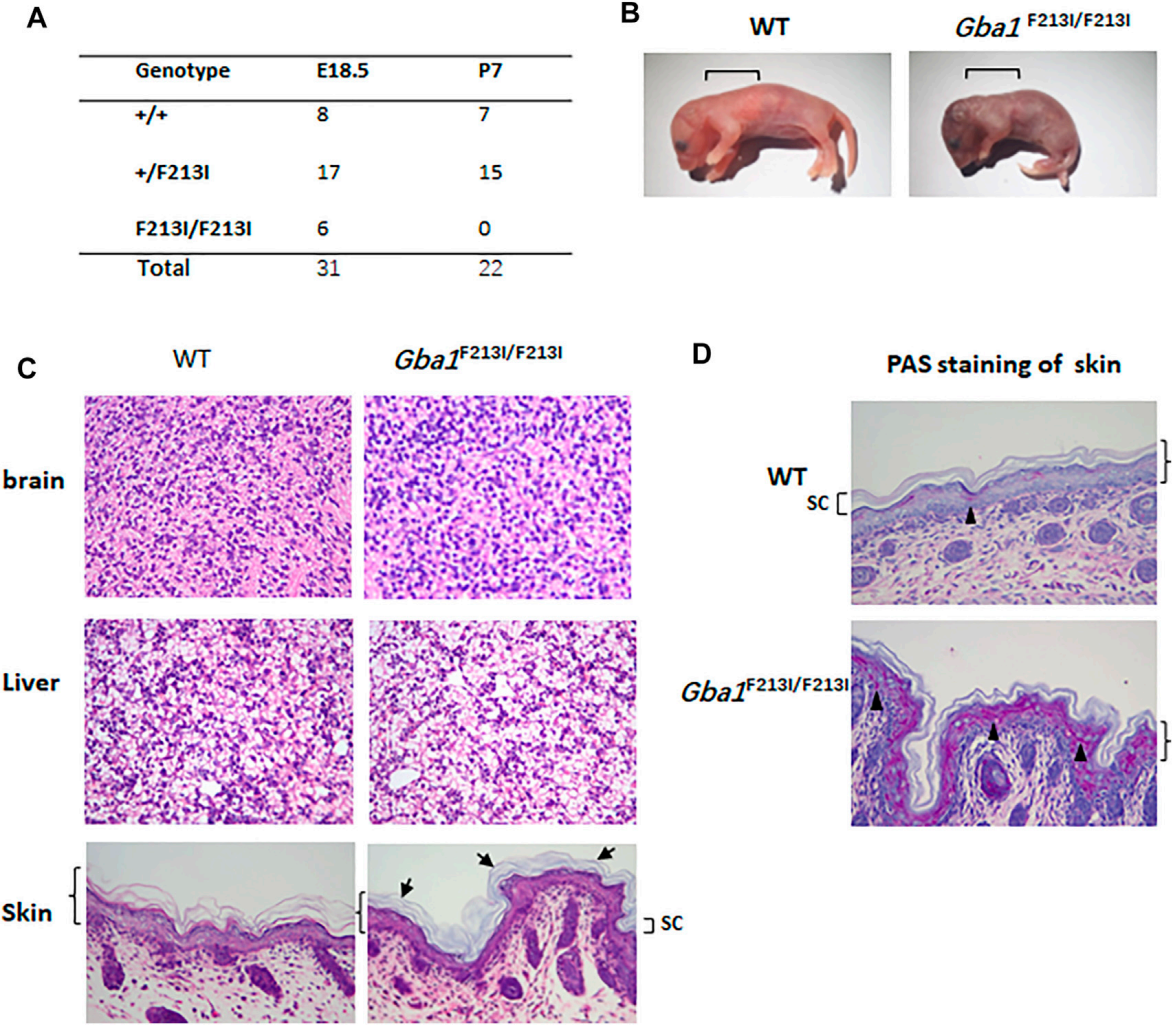
Mice with homozygous F213I mutation died postnatally within 24 h and displayed abnormal epidermis structure. **(A)** The number of embryos at E18.5 and the number of neonates at P14. Homozygotes for the F213I mutation died within 24 h after birth. **(B)** Photographs of the mice within 12 h after birth. Note the smaller size and wrinkled skin of the F213I mouse. The square brackets indicate the sites where skin samples were taken for the photomicrographs in C. **(C)** H&E images of brains, livers and epidermis. Tissues were collected from F213I homozygotes and wildtype littermate controls within 12 h after birth and processed for staining. The angle bracket indicates the epidermal layer. No significant differences are noted in the three other layers of the epidermis (stratum basal, stratum spinosum, and stratum granulosum) or the dermis. Stratum corneum (SC) was indicated by square basket. SC of newborn *Gba1* ^F213I/F213I^ mice was more basophilic, and more compact between layers, which was indicated by arrowheads. **(D)** PAS images of epidermis (×150). The angle bracket indicates the epidermal layer. Stratum corneum (SC) was indicated by square basket. Black triangles are used to highlight PAS-positive granules. PAS-positive granules in the granular and spinous layers were more prominent in the F213I mice than in control mice.

Genotype and survival statistics on offspring of *Gba1* ^+/F213I^ mice were collected (Figure 3A). Homozygous mh*Gba1*-F213I mutant mice were severely affected with small body size and turgor, red, and wrinkled appearance (Figure 3B). Sprinkling water to increase the humidity of the cage can prolong their survival time, and H&E staining of brain, liver and skin showed that no obvious Gaucher cells were found in available living *Gba1* ^F213I/F213I^ mice at P0, which was similar to the L444P mice (Liu et al., 1998). In the skin of *Gba1* ^F213I/F213I^ mice and inbred controls, all the four layers—basal (stratum basal), spinous (stratum spinosum), granular (stratum granulosum), and cornified (stratum corneum)—were identified in the epidermis. The *Gba1* ^F213I/F213I^ cornified layer appeared abnormal organization. Compared with wildtype littermate controls, the stratum corneum of newborn *Gba1* ^F213I/F213I^ mice was more compact between layers and more basophilic (Figure 3C). The Periodic Acid–Schiff (PAS) carbohydrate stain is a method that can detect glycolipids accumulation including glucosylceramide (Bogoeva and Petrusevska, 2001; Farfel-Becker et al., 2014), and the result showed that PAS-positive granules in the granular and spinous layers were more prominent in *Gba1* ^F213I/F213I^ mice (Figure 3D).

### GCase Activity Decreased in F213I Mutation Mice

**FIGURE 4:**
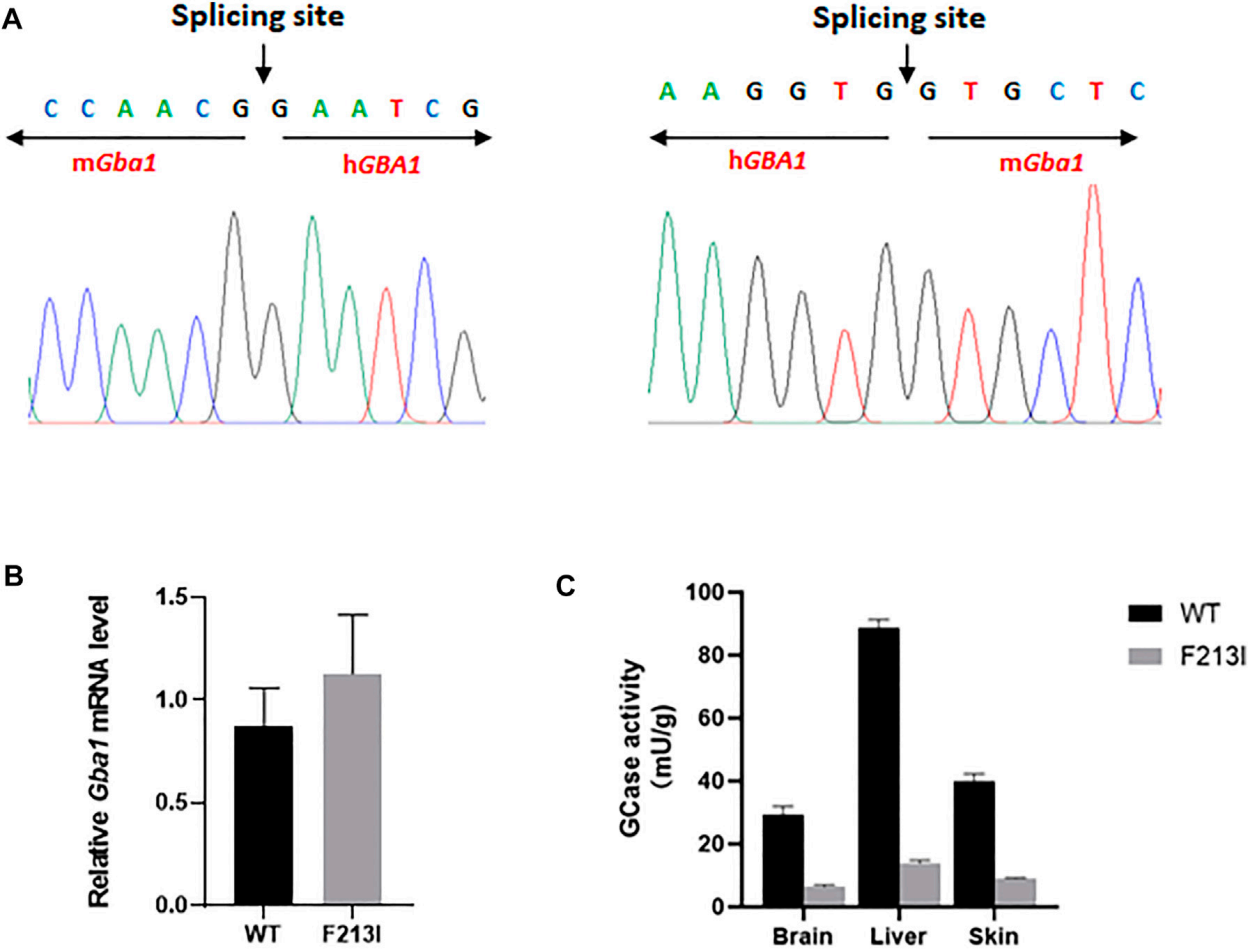
GCase activity decreased in F213I mutation mice. **(A)** Sequencing results of the splice sites of mh*Gba1*-F213I mRNA. The RNA was isolated from the brain of *Gba1* ^F213I/F213I^ mice on P0 day and reverse-transcripted. The splice sites were amplified by PCR and sequenced. The result showed that the partially humanized mh*Gba1*-F213I gene was correctly spliced. **(B)** qRT-PCR analysis of mRNA extracted from *Gba1* ^F213I/F213I^ mice (*n* = 3) and wild type mice (*n* = 3) at embryonic day 17, revealing no significant mRNA expression difference between *Gba1* ^F213I/F213I^ mice and wild type mice. Results were expressed as the mean—standard error of the mean (SEM) and the difference between the groups was analyzed by Student’s *t*-test. **(C)** GCase activity was detected in the skin, brain and liver of *Gba1* ^F213I/F213I^ mice (*n* = 3) on P0 day, using wild type mice (*n* = 3) as controls. The results were expressed as the mean—SEM and difference between groups was analyzed by Student’s *t*-test, ***p < 0.001. ****p < 0.0001.

cDNA from *Gba1* ^F213I/F213I^ mice was sequenced to confirm the correct splicing of the partially humanized *Gba1* mRNA, as designed (Figure 4A). *Gba1* mRNA expression in E17 whole embryos was measured by using quantitative reverse-transcription polymerase chain reaction (qRT-PCR), and the results showed that the expression of *Gba1* transcripts had no significant difference between *Gba1* ^F213I/F213I^ mice and wild type mice (Figure 4B). A large reduction in GCase activity was found in extracts from skin, liver and brain from *Gba1* ^F213I/F213I^ mice. Activity in the F213I mouse tissues was about 20% of normal controls, consistent with the values of clinic GD patients carrying the F213I mutation (Figure 4C).

## Discussion

GD is a common lysosomal storage disease in humans. It is caused by mutations in the gene (*GBA1*) coding for GCase, which lead to the accumulation of glucosylceramide in lysosomes (Stirnemann et al., 2017). There are nearly 500 types of allele variants reported in *GBA1*. Due to the distribution and diversity of human genetic variation, the frequency of each mutant allele varies. Several genetically modified GD mouse models have been established. The first GCase-deficient mouse was created by insertion of a neomycin resistance gene into *Gba1* gene, and died shortly after birth (Tybulewicz et al., 1992). Inducible *Gba1* deletion mouse models, in which the exons 9–11 are flanked by LoxP sites, was generated in 2006 (Enquist et al., 2006; Du et al., 2019). To simulate the gene mutation of GD patients, some *Gba1* point mutation GD mouse models were developed, such as L444P mice, D409V mice, and RecNciI (L444p and A456P double mutation) mice (Strasberg et al., 1994; Liu et al., 1998; Weber et al., 2021). F213I allele is the second high-frequency point mutation of *GBA1* gene in Chinese GD patients. So far, there have been few studies and no animal model on this allele. Therefore, to investigate the function of F213I allele and develop a model for future therapy by gene editing, we constructed a GD mouse model with partially humanized *Gba1* F213I allele.

Previous studies showed that it was feasible for conserved genes to exchange corresponding conserved exons to generate human-mouse chimeric gene without affecting the gene function. *GBA1* gene is highly conserved in human and mouse. In this research, the mouse *Gba1* exon 5-7 was replaced by the human counterparts to generate the human-mouse chimeric *Gba1* gene (mh*Gba1*) and *in vitro* GCase assays showed that the partial humanization had little effect on the activity of GCase, while the F213I mutation in mh*Gba1* (mh*Gba1*-F213I) greatly reduced the activity. To generate the mh*Gba1*-F213I mice, the mouse genomic fragment containing exons 5-7 were replaced by human corresponding region carrying F213I mutation, and the correctly spliced mature mRNA of mh*Gba1*-F213I was generated. GCase activity assay revealed that the GCase activity decreased in both central and peripheral tissues of *Gba1* ^F213I/F213I^ mice. However, like the previously reported *Gba1* point mutation mice, homozygous *Gba1* ^F213I/F213I^ mice died within 24 h after birth (Liu et al., 1998). So far, GD patients homozygous for N370S (p.Asn370Ser) or L444P mutations have been reported, and F213I was only found in compound heterozygote forms with N370S or L444P, but skin abnormalities were not diagnosed in these GD patients (Koprivica et al., 2000; Choy et al., 2007). Our results showed that the F213I homozygotes died within 24 h of birth and had red, wrinkled, dry skin that was indicative of disruption of the skin permeability barrier, resembling the phenotypes of mice homozygous for *Gba1* knockout, L444P or N370S mutation (Tybulewicz et al., 1992; Liu et al., 1998; Xu et al., 2003). Saposin C enhances GCase activity and protects GCase from intracellular proteolysis, *Gba1* ^D409V/D409V^:Saposin C^null/null^ mice also displayed similar skin phenotypes (Liou et al., 2019). As explained by Liu, Y., epidermal abnormalities were not observed in Gaucher patients, which may arise from the differences in skin barrier formation during fetal development (Liu et al., 1998). In rodents a competent skin permeability barrier forms very late in gestation 1–2 days before birth, while the permeability barrier normally forms well before birth at between 30 and 34 weeks of gestation in human, which provides enough time for residual GCase mediated conversion of glucosylceramide to ceramide during this period to produce a competent barrier (Holleran et al., 1994; Kalia et al., 1998; Doering et al., 2002). However, the residual level of GCase activity in F213I mice may be insufficient to completely process the epidermal glucosylceramide in this short time period during gestation. Infants with less residual level of GCase activity have been described to have a severe skin phenotype (Sidransky et al., 1996). Another factor contributing to the glucosylceramide storage in epidermis but not in brain and liver in the F213I mouse could be related to biochemical differences of the glucosylceramides found in different tissues (Liu et al., 1998), for example skin contains glucosylceramides with additional hydroxyl groups and with very long chain fatty acids, in addition to common types of glucosylceramides like those found in brain and liver (Wertz, 1992). The F213I mutation may render the GCase enzyme less active against the hydroxylated glucosylceramides with long chain fatty acids than against the common types of glucosylceramides, resulting in storage restricted to epidermis.

Enzyme replacement therapy (ERT) and substrate reducing therapy (SRT) are used as clinical therapeutic methods, and AAV-mediated gene addition has been investigated by other researchers and our team (Du et al., 2019; Hurvitz et al., 2019; Jackson et al., 2019; Peng et al., 2021). Gene editing or repairing could be an alternative treatment method for GD disease, but no researches have been conducted. Because this mh*Gba1*-F213I mice model has the human genomic DNA around F231I mutation site in mouse *Gba1* allele, it will be suitable to detect Crispr/CAS9-mediated repairing of human *GBA1*-F213I mutation in this model. And as we described previously, Ubc-CreERT2-induced global *Gba1* knockout (*Gba1* ^Flox/Flox^:Ubc-CreERT2 mice) can solve the problem of early postnatal death (Du et al., 2019). It will be interesting to detect whether *Gba1* ^F213I/Flox^:Ubc-CreERT2 mice have extended survival time and can be used for future gene edition therapy. Several researches have reported that the carbohydrate mimic N-octyl-β-valienamine (NOV) up-regulated cellular enzyme activity of some GCase mutants in cultured GD fibroblasts, including F213I, N188S, G202R and N370S (Lin et al., 2004; Lei et al., 2007; Luan et al., 2010), so it will be interesting to detect the therapeutic effects of NOV in *Gba1* ^F213/F213I^ mice. Because F213I homozygotes die early after birth, it will be feasible to treat pregnant mice and detect its effects on the pups and enzyme activity.

Now, it is clear that the presence of *GBA1* mutation in homozygous or heterozygous form is associated with an approximately 20-fold increase in the risk for Parkinson disease (PD) (Schapira, 2015; Gegg and Schapira, 2018; Do et al., 2019). F213I was the second most common mutation in patients with Gaucher disease (14%), but its mutation frequency was relatively low (only 2% of the pathogenic variants in patients with PD) (Mitsui et al., 2009; Sun et al., 2010). Up to now, we have not observed the symptoms of Parkinson disease in 6-month-old F213I heterozygotes. A lifespan observation of the F213I mice may be needed in future research.

Moreover, *GBA1* has a pseudogene *GBAP1*, acting as competing-endogenous RNA (ceRNA) to regulate *GBA1* expression (Straniero et al., 2017). It is possible that *GBAP1* is involved in the pathogenesis of PD and GD, and manipulation of *GBAP1* may have potential therapeutic effects on the diseases. A research also designed specific easy-to-use CRISPR-Cas9 gene editing strategy to correct the common *GBA1* N370S mutation and to ensure the integrity of this pseudogene (Hanss et al., 2019). Mice lack the pseudogene that is present in humans and apes, so we should consider this deficiency when using GD mouse models, for example it cannot be excluded that the absence of the pseudogene in mice may affect the manifestation of PD or GD symptoms.

In summary, our research revealed that F213I mutation caused early postnatal lethality and this partially humanized mouse GD model has the potential for future gene repairing researches *in vivo*.

## Data Availability

The raw data supporting the conclusions of this article will be made available by the authors, without undue reservation.
