# Supplementary material for: Establishment and Phenotypic Analysis of the Novel Gaucher Disease Mouse Model With the Partially Humanized Gba1 Gene and F213I Mutation
Source: Front Genet. 2022 May 27;13:892457. doi: 10.3389/fgene.2022.892457 (PMC9196271; doi:10.3389/fgene.2022.892457)
Supplement: Supplementary file 1 [file Table1.docx]

**Supplementary Table S1 Sequences of related PCR primers.**

| **Primer** | **5rime** |
| --- | --- |
| **m*Gba1*-F** | TAGCAAGCCCAGTTCACACA |
| **m*Gba1*-T** | GAGTAGGCGGACATTGTGGT |
| **h*GBA1*-F** | GTCAATCTTGCCCACTCCAT |
| **h*GBA1*-T** | GAGTAGGCGGACATTGTGGT |
| **m*Gapdh*-RF** | TGTCGTGGAGTCTACTGGTGTCTT |
| **m*Gapdh*-RT** | TTCTCGTGGTTCACACCCATCACA |
| **m*Gba1*-RF** | TGGGTACCTTCAGCCGTTAC |
| **m*Gba1*-RT** | CGCCTCCAAATCCTTTCAC |
| **m*Gba1*-5F** | TAGCAAGCCCAGTTCACACA |
| **h*GBA1*-5T** | GGAACCAAATGTCAGGGATG |
| **h*GBA1*-3F** | AACGTGTTGAAACCCTGTGTC |
| **m*Gba1*-3T** | AGCACCCACACCTATGCAA |
